# D3O-IIoT: deep reinforcement learning-driven dynamic deception orchestration for industrial IoT security

**DOI:** 10.1038/s41598-025-33426-4

**Published:** 2025-12-21

**Authors:** Usman Wushishi, Altaf Hussain, Muhammad Imran Khalid, Nasir Hussain, Mona Jamjoom, Zahid Ullah

**Affiliations:** 1 School of Computer Science and Technology, Chongqing University of Posts and Telecommunications, Chongqing, 400065 China; 2 Department of Computer Sciences, College of Computer and Information Sciences, Princess Nourah Bint Abdulrahman University, Riyadh, 11671 Saudi Arabia; 3 Information Systems Department, College of Computer and Information Sciences, Imam Mohammad Ibn Saud Islamic University (IMSIU), Riyadh, 11432 Saudi Arabia

**Keywords:** Deep reinforcement learning, Industrial IoT security, Dynamic deception, Cyber defense, Attack mitigation, Energy science and technology, Engineering, Mathematics and computing

## Abstract

The industrial Internet of Things (IIoT) systems are under mounting cyber threats that take advantage of the resource shortage and operational vulnerability of industrial systems. The current intrusion detection schemes are based on either the static or passive form of defense that is not dynamically adapted to the changing attacks. This paper presents D3O-IIoT, a progressive reinforcement learning model that dynamically coordinates deception techniques, including honeypot deployment, moving target defense, fake telemetry injection, and node isolation on the basis of real time threat monitoring. The defense problem is formulated as a Markov Decision Process, in which a Dueling Deep Q-Network agent maximizes a multi-objective reward to balance between attack mitigation, deception engagement, false positive control and resource cost. Experiments on three IIoT datasets (CIC-IIoT2025, WUSTL-IIoT2021, TON-IoT) demonstrate that D3O-IIoT has a 13.7% attack mitigation rate with a 0.3% false alarm, which is an improvement of 293–767% (*p* < 0.0001) over baselines. Generalization is confirmed by cross-dataset validation (97.7% and 77.8% retention on TON-IoT and WUSTL-IIoT, respectively). Results of Ablation determine that the most critical component of reward is false positive control (51.4% degradation upon removal) and that sensitivity analysis indicates the possibility of 46.1% tunability through risk threshold change. The acquired policy favors isolation (71.2 per cent) on confirmed threats and honeypots (15.4 per cent) on reconnaissance with a 2.07ms latency that can be deployed in real time. D3O-IIoT builds upon IIoT cybersecurity by substituting fixed set rule-based defenses with dynamic and learning-based deception orchestration, balancing various practical goals under resource-constrained conditions.

## Introduction

The IIoT systems have been established as a central part of modern manufacturing, energy, and vital infrastructures operation processes^1^. These cyber-physical networks combine IT systems and operational technology to provide real-time and predictive control. Nevertheless, this interconnectedness opens the industrial settings to cyber threats that can disrupt the production process, compromise the integrity of safety, or steal confidential information^2^. The Colonial Pipeline ransomware attack and the Ukrainian power grid break-in are the high-profile examples of the devastating consequences of IIoT security breach, and a call to the creation of more adaptive and proactive security measures. The traditional intrusion detection systems (IDS) implemented to monitor enterprise networks are insufficient in IIoT networks^3^.

Industrial devices have severe computational and energy limitations, and traditional communication standards (e.g., Modbus, DNP3, OPC-UA) are not based on enterprise traffic. These considerations make heavy-weight machine learning defenses or signature-based inappropriate. Furthermore, the industrial processes require close to zero false alarms, because the misidentified automated actions may lead to expensive or hazardous operational disruptions^4^. The majority of current IIoT defenses are based on passive detection based on anomaly or classification models^5^. Although they work with known attacks, these systems do not dynamically adapt and respond to emerging threats. Similarly, rule or static deception systems are not flexible to change tactics based on attacker behavior. Game-theoretic models simulate adversarial interactions, and are computationally intractable in real-time as well as unable to learn continuously as demonstrated by real-world systems such as human behavior or artificial intelligence^6^. Reinforcement learning (RL) can present a successful basis of adaptive cybersecurity by learning the best policy of sequenced decisions via interaction with the environment^7^.

Nonetheless, its use in IIoT defense presents problems of trade-offs between various goals (attack mitigation, precision, cost), high-dimensional state representations, and low decision latency in real-time of decision-making^8^. As a means to overcome these shortcomings, this paper introduces D3O-IIoT (Deep Reinforcement Learning-Driven Dynamic Deception Orchestration for Industrial IoT), a model that develops IIoT defense as a Markov Decision Process. A Dueling Deep Q-Network agent is trained to design deception plans, such as honeypot deployment, moving target defense, fake telemetry injection and node isolation, depending on the real-time threat observance. Multi-objective rewarding function is used to balance adaptively the mitigation effectiveness, deception interaction, false positive control and the cost of resources. The main contributions of the work are as follows:

- We suggest D3O-IIoT which is a deep reinforcement learning model for dynamically orchestrating deception in IIoT setting. The framework learns adaptive defense policies that balance attack mitigation, precision control, and resource efficiency through multi-objective reward optimization.
- We design a Dueling Deep Q-Network architecture with 22-dimensional IIoT state representation capturing network, protocol, and telemetry features, achieving 2.07ms decision latency suitable for real-time industrial deployment.
- We perform extensive testing on three real-world IIoT datasets, namely CIC-IIoT2025, WUSTL- IIoT2021, and TON-IoT, with 13.7% attack mitigation rate and 0.3% false alarm rate, and 293–767% improvement over six baselines with significant statistical values (*p* < 0.0001).
- Extensive analysis, such as cross-dataset validation, ablation, and sensitivity analyses, prove that D3O-IIoT has strong generalization, interpretable component contributions, and tunable deployment behavior, which makes it viable in many IIoT security settings.

The rest of this paper will follow the following structure: Sect. “Related work” is a review of related literature in IIoT intrusion detection, deception-based defense, and reinforcement learning in cybersecurity. Section “Methodology” provides the description of the D3O-IIoT approach, which comprises the formulation of the problem, the design of the multi-objective rewards, the Dueling DQN model, and the training process. Section “Experimental setup and results” includes dataset preprocessing, training convergence, optimization, and experimental findings, Sect.“Discussion” explains implications and practical implementation issues, and Sect. “Conclusion” concludes the paper.

## Related work

The literature applied to D3O-IIoT lies within three primary areas, including intrusion detection in the Industrial IoT, deception-based defenses, and reinforcement learning in adaptive cybersecurity. This part summarizes main developments and outlines existing constraints that drive the suggested framework.

### Intrusion detection in industrial IoT

The initial IIoT intrusion detection systems (IDS) used signature- or rule-based models which were inherited by the traditional IT security^9^. Although useful when dealing with known threats, these methods cannot identify zero-day attacks or polymorphic attacks since they rely on handcrafted signatures. This was later followed by anomaly-based IDS such as statistical profiling^10^ and classical machine learning algorithms like SVMs, random forests and k-nearest neighbors^11^ in order to enhance the detection of hidden threats. Nevertheless, they do not perform well in dynamic industrial settings where data distributions are non- stationary, and the ratio between benign and attack is not balanced. Convolutional and recurrent neural networks are deep learning algorithms that have shown better detection rates on the benchmark datasets like TON-IoT and CIC-IDS datasets^12,13^. A further improvement in the extraction of features on multivariate sensor data is through hybrid architectures that comprise of autoencoders and graph neural networks^14,15^. However, with these capabilities, a majority of deep IDS models are passive and only able to detect and not respond. Additionally, they usually need retraining of new network topologies which limits scalability over heterogeneous IIoT deployments^16^. Recent developments involve multi-objective optimization methods that combine deep learning with metaheuristic feature selection and almost perfect results on benchmark data sets are obtained^44^, but they are limited to detection instead of active response.

### Deception-based defense mechanisms

The goal of deception technologies is to deceive attackers and raise the cost of attacks, using traps, decoys, or dynamically evolving network settings^17^. Honeypots and honeynets have been used for a long time to record attacker activity^18^ moving target defense (MTD) distorts network properties like IP addresses or routes to minimize the success of reconnaissance by the attacker^19^. Fake telemetry injection and decoy services enhance this approach by placing false data within industrial monitoring networks^20^. Nevertheless, the vast majority of the available systems of deception implement a static or rule-based arrangement, which triggers predefined responses regardless of the changing attack conditions^21^. The approaches of game-theoretic deception models somewhat involve adaptivity because payoff structures are known in advance and the interactions between attackers and defenders are modeled as strategic games which are computationally infeasible in large IIoT systems. As such, these systems are unable to learn continuously or adapt according to real-time feedback of ongoing attacks^22^. Recent literature has investigated game-theoretic models of honeypot implementation in industrial systems^42^ and adaptive moving target defense strategies against DDoS attacks in IIoT have shown better resource availability and reaction periods^43^ but still do not have combined learning-based coordination.

### Reinforcement learning for cybersecurity

Reinforcement learning (RL) is a type of learning which presents the adaptive decision-making process by interacting with the environment continuously^23^. Initial applications in cybersecurity are to optimize the parameters of IDSs^7^, or to automate the choice of response in a network defense simulation^24^. Recent deep RL models, including Deep Q-Networks (DQN), policy-gradient, and actor-critic, have been used to classify traffic, detect anomalies, and optimize moving target defenses^25–27^. However, the vast majority of RL-based defenses are based on single-objective optimization, like detection accuracy or latency and do not take the trade-offs between attack mitigation, false positive control, and resource cost into account. There are a limited number of works that feature multi-objective reward frameworks that best suit IIoT where operational accuracy is as important as the coverage of the detection. Furthermore, a great number of studies use simplified network simulations covering industrial telemetry, and therefore, not applicable to real-world IIoT environments^28^. Recent research has shown that value-based DRL approaches are effective, with DDQN achieving 99.7% of accuracy on industrial control system datasets^40^, and hybrid DRL-IDS systems reaching 99.85% of detection accuracy and response times of sub-1500ms^41^. The methods, however, are strongly oriented on the detection accuracy without combining active deception orchestration.

A number of representative methods have been taken as baseline in order to contextualize and assess performance of the proposed framework. The Static RL Deception models^29^ use constant deception probabilities without an adaptive learning approach and, therefore, have limited responsiveness to changing attack patterns. DRL without Deception^30^ adopts reinforcement learning in detecting intrusion but omits active deception, hence it does not adopt methods of engagement against attackers when detected. Game- theoretic policies^31^ model the interaction of defenders and attackers in an equilibrium framework but are computationally complex and unable to adapt online. IDS-only systems^32^ include those that only use passive detection and are a constraint on the extent of mitigation capability and Adaptive DL-Deception^33^ are based on deep learning-based thresholds to choose actions but is reactive and not sequential. Lastly, Random Policy^34^ performs random actions, giving a stochastic baseline on which lower performance can be compared. All these baselines reflect the current range of IIoT defensive paradigms between passive detection and adaptive but fixed deception, and represent the relative basis on the effectiveness of D3O-IIoT at the same conditions of evaluation.

### Research gap and motivation

There is a continuous gap between adaptive detection and dynamic deception as described in the reviewed literature. Passive deep IDS have high detection rate but are incapable of preventing active threats. On the other hand, deception mechanisms augment workload on adversaries but have no data-driven coordination. Game-theoretic and RL-based frameworks have already started to overcome this gap, but today, models are still confined to single-objective models, fixed reward functions, or computational infeasibility in resource- constrained IIoT settings. Table 1 provides an overview of the capabilities of new methods, showing that none of the methods use RL-based learning, active deception, multi-objective optimization, and design-specific to IIoT. D3O-IIoT solves these issues by combining deep reinforcement learning with orchestration of defenses using deception. It uses a Dueling Deep Q-Network and multi-objective rewards to discover how to achieve attack mitigation success, false positive accuracy and cost in real time. This combination brings current literature to a stage of complete autonomy, context awareness, and operationally feasible IIoT security models.

**Table 1:** Comparison of D3O-IIoT with recent IIoT security approaches.

Method	RL	Deception	Multi-Obj.	Adaptive	IIoT
Sangoleye et al^40^.	✓	–	–	✓	✓
Kanimozhi & Ramesh^41^	✓	–	–	✓	–
Peters & Gkoktsis^42^	–	✓	–	–	✓
Swati et al^43^.	–	✓	–	✓	✓
Asgharzadeh et al^44^.	–	–	✓	–	✓
**D3O-IIoT (Ours)**	✓	✓	✓	✓	✓

## Methodology

### Problem formulation

**Fig. 1:**
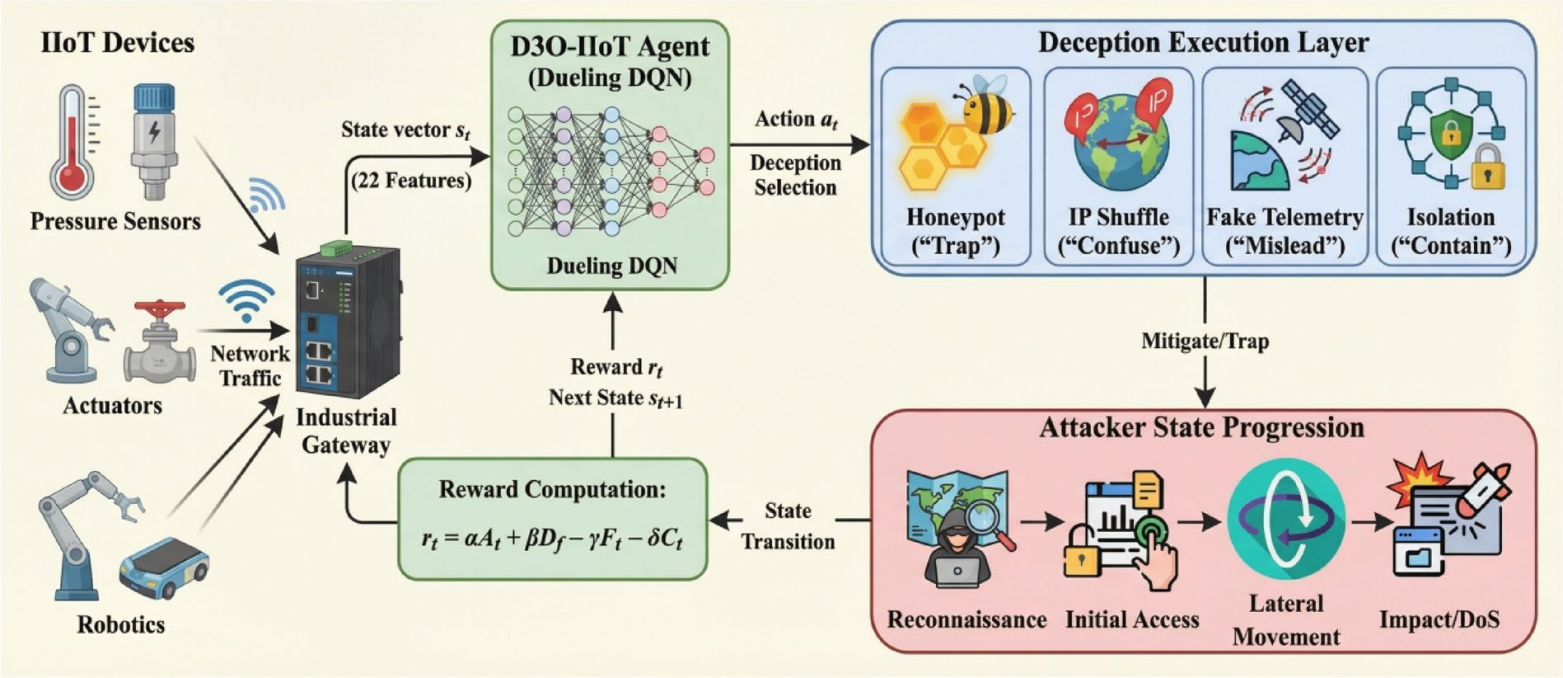
Overall D3O-IIoT system architecture.

The Industrial IoT defense problem is formulated as a Markov Decision Process (MDP) where an autonomous agent learns optimal deception strategies through interaction with a simulated IIoT network environment. The MDP is defined by the tuple $\:\mathcal{M}=(\mathcal{S},\mathcal{A},\mathcal{P},\mathcal{R},\gamma\:)$, where $\:\mathcal{S}$ represents the state space encoding network conditions and threat indicators, $\:\mathcal{A}$ denotes the discrete action space of deception tactics, $\:\mathcal{P}:\mathcal{S}\times\:\mathcal{A}\times\:\mathcal{S}\to\:[0,1]$ defines state transition probabilities, $\:\mathcal{R}:\mathcal{S}\times\:\mathcal{A}\to\:\mathbb{R}$ specifies the reward function, and $\:\gamma\:\in\:[0,1]$ is the discount factor for future rewards. Figure 1 illustrates the complete framework architecture.

At each time step $\:t$, the agent observes state $\:{s}_{t}\in\:\mathcal{S}$, selects deception action $\:{a}_{t}\in\:\mathcal{A}$ according to policy $\:\pi\:\left({a}_{t}\right|{s}_{t})$, receives reward $\:{r}_{t}=\mathcal{R}({s}_{t},{a}_{t})$, and transitions to next state $\:{s}_{t+1}\sim\:\mathcal{P}(\cdot\:|{s}_{t},{a}_{t})$. The objective is to learn optimal policy $\:{\pi\:}^{\mathrm{*}}$ that maximizes expected cumulative discounted reward:

1 $$\:{\pi\:}^{\mathrm{*}}=\mathrm{arg}\underset{\pi\:}{\mathrm{max}}{\mathbb{E}}_{\pi\:}\left[\sum\limits_{t=0}^{{\infty\:}}{\gamma\:}^{t}{r}_{t}\right]\:\:\:\:\:$$

The state space $\:\mathcal{S}\subset\:{\mathbb{R}}^{22}$ comprises normalized features extracted from network flow records and IIoT telemetry. Each state vector $\:{s}_{t}=[{f}_{1},{f}_{2},\dots\:,{f}_{22}{]}^{T}$ contains network statistics (flow duration, packet counts, byte volumes), protocol information (TCP flags, port numbers), temporal characteristics (inter-arrival times), and system metrics (CPU usage, latency). All features are normalized via min-max scaling (Eq. 2) to $\:[0,1]$ range:

2 $$\:f{{\prime\:}}_{i}=\frac{{f}_{i}-{f}_{i,\mathrm{min}}}{{f}_{i,\mathrm{max}}-{f}_{i,\mathrm{min}}}\:\:\:\:\:$$

where normalization parameters $\:({f}_{i,\mathrm{min}},{f}_{i,\mathrm{max}})$ are computed exclusively from training data to prevent information leakage.

The action space $\:\mathcal{A}=\{{a}_{1},{a}_{2},{a}_{3},{a}_{4},{a}_{5}\}$ consists of five deception tactics: $\:{a}_{1}$ (honeypot deployment) diverts attackers to isolated decoy systems, $\:{a}_{2}$ (IP shuffling via Moving Target Defense) obfuscates network topology, $\:{a}_{3}$ (fake telemetry injection) misleads reconnaissance, $\:{a}_{4}$ (node isolation) contains compromised systems, and $\:{a}_{5}$ (no-operation) maintains current state when intervention is unnecessary.

Attack detection employs a risk scoring mechanism (Eq. 3) that gates deception action deployment. Given state $\:{s}_{t}$, the risk score is computed as:

3 $$\:\rho\:\left({s}_{t}\right)=0.3\cdot\:{f}_{\mathrm{pkt\_rate}}+0.25\cdot\:{f}_{\mathrm{flow\_bytes}}+0.25\cdot\:{f}_{\mathrm{syn\_flags}}+0.2\cdot\:|{f}_{\mathrm{avg\_pkt}}-0.5|\:\:\:\:\:\:$$

where coefficients weight features based on their attack-indicative strength. Deception actions are triggered when $\:\rho\:\left({s}_{t}\right)>\tau\:$, with threshold $\:\tau\:=0.470$ determined through validation set optimization.

### D3O-IIoT system architecture

The IIoT environment generates 22-dimensional state vectors capturing network, protocol, and telemetry features. The Dueling DQN agent observes the current state, selects an optimal deception action (honeypot, IP shuffling, fake telemetry, node isolation, or no-operation), and executes it in the defense environment. The environment computes a multi-objective reward based on attack mitigation, deception engagement, false positives, and resource cost, which guides continuous policy refinement.

### Multi-objective reward function

The reward function (Eq. 4) balances four competing objectives: attack mitigation effectiveness, deception engagement duration, false positive minimization, and resource cost control. At time step $\:t$, the reward is computed as:

4 $$\:{r}_{t}=\alpha\:\cdot\:{A}_{t}+\beta\:\cdot\:{D}_{t}-\gamma\:\cdot\:{F}_{t}-\delta\:\cdot\:{C}_{t}\:\:\:\:\:$$

where $\:{A}_{t}\in\:\{0,1\}$ indicates successful attack mitigation, $\:{D}_{t}\in\:[0,1]$ measures normalized attacker engagement time in deception environments, $\:{F}_{t}\in\:\{0,1\}$ penalizes false positive detections, and $\:{C}_{t}\in\:[0,1]$ represents normalized deception deployment cost. The weight vector $\:[\alpha\:,\beta\:,\gamma\:,\delta\:]=[0.70,0.20,0.06,0.04]$ was optimized through systematic grid search over candidate configurations. The mitigation of attacks is given the first-order importance as the main goal and lower values of the false positive and cost penalties allow aggressive defense. This allocation is confirmed by ablation analysis (Sect. “Ablation study”).

Attack mitigation success $\:{A}_{t}$ is determined by comparing attacker state transitions against deception action outcomes. The attacker state $\:{\sigma\:}_{t}\in\:\varSigma\:$ evolves through phases: $\:{\sigma\:}_{\mathrm{idle}}$, $\:{\sigma\:}_{\mathrm{recon}}$, $\:{\sigma\:}_{\mathrm{access}}$, $\:{\sigma\:}_{\mathrm{lateral}}$, $\:{\sigma\:}_{\mathrm{impact}}$, with terminal states $\:{\sigma\:}_{\mathrm{trapped}}$, $\:{\sigma\:}_{\mathrm{confused}}$, $\:{\sigma\:}_{\mathrm{contained}}$, $\:{\sigma\:}_{\mathrm{neutralized}}$, and $\:{\sigma\:}_{\mathrm{success}}$. Successful mitigation occurs when deception actions force transitions to defensive terminal states, formalized as:

5 $$\:{A}_{t}=\left\{\begin{array}{ll}1&\:\mathrm{if\:}{\sigma\:}_{t+1}\in\:\{{\sigma\:}_{\mathrm{trapped}},{\sigma\:}_{\mathrm{confused}},{\sigma\:}_{\mathrm{co}\mathrm{ntained}},{\sigma\:}_{\mathrm{neutralized}}\}\:\:\:\:\:\\\:0&\:\mathrm{otherwise}\end{array}\right.$$

Engagement duration $\:{D}_{t}$ quantifies the effectiveness of honeypot and misdirection tactics in prolonging attacker dwell time within monitored environments, computed as:

6 $$\:{D}_{t}=\frac{{t}_{\mathrm{engage}}}{{t}_{\mathrm{max}}}\:\:\:\:\:$$

where $\:{t}_{\mathrm{engage}}$ is the cumulative time the attacker remains in trapped or confused states, and $\:{t}_{\mathrm{max}}=25$ is the maximum observed engagement duration during training. This normalization ensures scale consistency across episodes.

False positive penalty $\:{F}_{t}$ activates when deception actions are applied to benign traffic, identified through ground truth labels:

7 $$\:{F}_{t}=\left\{\begin{array}{ll}1&\:\mathrm{if\:}{a}_{t}\ne\:{a}_{5}\wedge\:{\mathrm{label}}_{t}=\mathrm{benign}\\\:0&\:\mathrm{otherwise}\end{array}\right.\:\:\:\:\:$$

where $\:{a}_{5}$ represents the no-operation action. This component proved critical in ablation studies, with its removal causing 51.4% performance degradation.

Deception cost $\:{C}_{t}$ accounts for computational and operational overhead of each action, defined as:

8 $$\:{C}_{t}=\left\{\begin{array}{lll}0.05&\:\mathrm{if\:}{a}_{t}={a}_{1}\mathrm{\:(honeypot)}\\\:0.03&\:\mathrm{if\:}{a}_{t}={a}_{2}\mathrm{\:(IP\:shuffle)}\\\:0.02&\:\mathrm{if\:}{a}_{t}={a}_{3}\mathrm{\:(fake\:telemetry)\:\:\:\:\:}\\\:0.08&\:\mathrm{if\:}{a}_{t}={a}_{4}\mathrm{\:(isolation)}\\\:0.001&\:\mathrm{if\:}{a}_{t}={a}_{5}\mathrm{\:(no-op)}\end{array}\right.$$

These values reflect relative operational impact: isolation (0.08) disrupts communications, honeypot (0.05) requires infrastructure, IP shuffling (0.03) and fake telemetry (0.02) impose moderate overhead, while no-operation (0.001) represents minimal monitoring cost. The low weight $\:\delta\:=0.04$ in Eq. 4 permits aggressive defense strategies while preventing wasteful action selection.

### Dueling deep Q-Network architecture

**Fig. 2:**
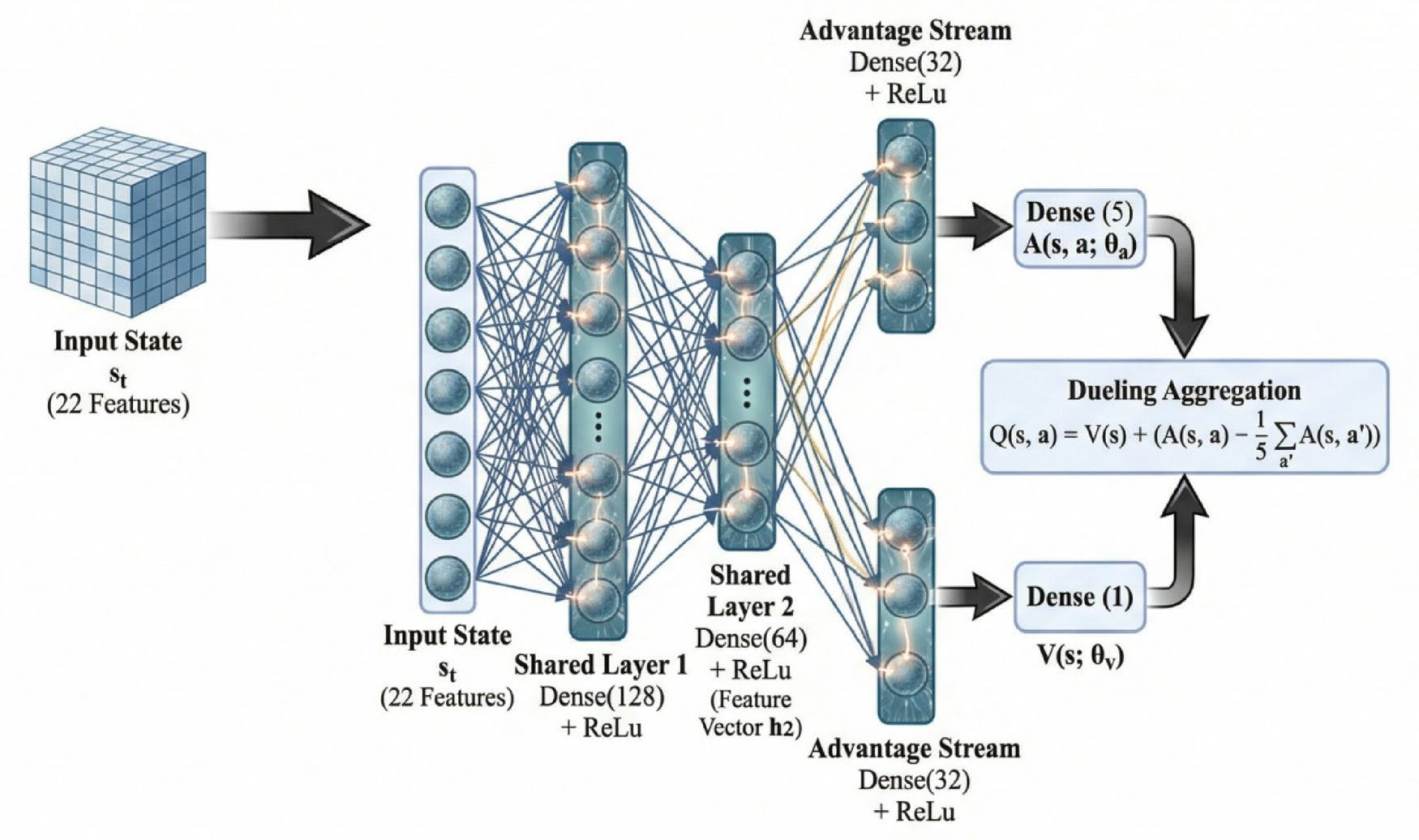
Dueling Deep Q-Network (DQN) architecture used in the D3O-IIoT framework. The input state vector $\:{s}_{t}\in\:{\mathbb{R}}^{22}$ passes through shared feature extraction layers (Dense 128 and Dense 64 with ReLU activations), producing latent representation $\:{h}_{2}$. The network then branches into two parallel streams: a value stream estimating the state value $\:V\left(s\right)$ and an advantage stream computing action-specific advantage $\:A(s,a)$. Both are combined through the dueling aggregation layer to produce final Q-values $\:Q(s,a)$ for the five deception actions, enabling epsilon-greedy exploration during training and optimal action selection during evaluation.

The agent employs a Dueling Deep Q-Network architecture (Fig. 2) that decomposes the state-action value function into state value and action advantage components. Intuitively, this separation allows the agent to learn “how good is this network state?” independently from “which deception action is best here?” For IIoT defense, this is advantageous because many network states require no intervention regardless of action choice, while threat states demand precise action selection. By learning these aspects separately, the agent converges faster and generalizes better across diverse traffic patterns. This decomposition enables more efficient learning by separating the value of being in a state from the relative advantage of each action in that state. The Q-function approximation is structured as:

9 $$\:Q(s,a;\theta\:)=V(s;{\theta\:}_{v})+\left(A(s,a;{\theta\:}_{a})-\frac{1}{\left|\mathcal{A}\right|}\sum\limits_{a\mathcal{{\prime\:}}\in\:\mathcal{A}}A(s,a{\prime\:};{\theta\:}_{a})\right)\:\:\:\:\:$$

where $\:V(s;{\theta\:}_{v})$ represents the value function estimating expected return from state $\:s$, $\:A(s,a;{\theta\:}_{a})$ denotes the advantage function quantifying how much better action $\:a$ is compared to the average action in state $\:s$, and $\:\theta\:=\{{\theta\:}_{v},{\theta\:}_{a}\}$ are learnable parameters. The advantage mean subtraction ensures identifiability by centering advantages around zero.

The network architecture consists of three components: a shared feature extraction pathway, a value stream, and an advantage stream. The shared pathway processes the 22-dimensional state vector through two fully connected layers with ReLU activations:

10 $$\:\begin{array}{rr}{h}_{1}&\:=\mathrm{ReLU}({W}_{1}s+{b}_{1}),\:{W}_{1}\in\:{\mathbb{R}}^{128\times\:22}\end{array}$$

11 $$\:\begin{array}{rr}{h}_{2}&\:=\mathrm{ReLU}({W}_{2}{h}_{1}+{b}_{2}),\:{W}_{2}\in\:{\mathbb{R}}^{64\times\:128}\:\:\:\:\:\:\:\:\:\:\:\:\:\:\:\:\:\:\:\:\:\:\:\:\end{array}$$

The value stream (Eq. 12) maps $\:{h}_{2}$ from Eq. 11 to scalar state value through a 32-neuron hidden layer:

12 $$\:V(s;{\theta\:}_{v})={w}_{v}^{T}\mathrm{ReLU}({W}_{v}{h}_{2}+{b}_{v}),\:{W}_{v}\in\:{\mathbb{R}}^{32\times\:64}\:\:\:\:\:\:\:\:\:\:\:\:\:\:\:\:$$

The advantage stream (Eq. 13) produces action-specific advantages through parallel architecture:

13 $$\:A(s,a;{\theta\:}_{a})={W}_{a}^{\left(a\right)}\mathrm{ReLU}({W}_{a}{h}_{2}+{b}_{a}),\:{W}_{a}\in\:{\mathbb{R}}^{32\times\:64}\:\:\:\:\:\:\:\:\:\:$$

where $\:{W}_{a}^{\left(a\right)}$ denotes the weight vector corresponding to action $\:a$. These components combine via Eq. 9 to produce final Q-values.

Training employs experience replay to break temporal correlations in sequential observations. Transitions $\:({s}_{t},{a}_{t},{r}_{t},{s}_{t+1})$ are stored in replay buffer $\:\mathcal{D}$ with capacity 150,000. At each training step, a minibatch of 64 transitions is sampled uniformly to compute the temporal difference loss:

14 $$\:\mathcal{L}\left(\theta\:\right)={\mathbb{E}}_{(s,a,r,s\mathcal{{\prime\:}})\sim\:\mathcal{D}}\left[{\left(r+\gamma\:\underset{a{\prime\:}}{\mathrm{max}}Q(s{\prime\:},a{\prime\:};{\theta\:}^{-})-Q(s,a;\theta\:)\right)}^{2}\right]\:\:\:\:\:\:\:\:\:\:$$

where $\:{\theta\:}^{-}$ represents target network parameters updated periodically via soft update rule:

15 $$\:{\theta\:}^{-}\leftarrow\:{\tau\:}_{\mathrm{target}}\theta\:+(1-{\tau\:}_{\mathrm{target}}){\theta\:}^{-}\:\:\:\:\:\:\:\:\:\:\:\:\:\:\:\:\:\:\:\:\:\:\:\:\:\:\:\:\:\:$$

with $\:{\tau\:}_{\mathrm{target}}=0.001$ controlling the update rate.

Exploration follows epsilon-greedy policy (Eq. 16) with exponential decay. Action selection probability is:

16 $$\:\pi\:\left(a\right|s)=\left\{\begin{array}{ll}\mathrm{arg}\underset{a\in\:\mathcal{A}}{\mathrm{max}}Q(s,a;\theta\:)&\:\mathrm{with\:probability\:}1-\epsilon\\\:\mathrm{Uniform}\left(\mathcal{A}\right)&\:\mathrm{with\:probability\:}\epsilon\end{array}\right.\:\:\:\:\:\:\:\:\:\:$$

where $\:\epsilon$ decays from initial value 1.0 to minimum 0.08 according to:

17 $$\:{\epsilon}_{t}=\mathrm{max}({\epsilon}_{\mathrm{min}},{\epsilon}_{0}\cdot\:{\lambda\:}^{t})\:\:\:\:\:\:\:\:\:\:\:\:\:\:\:\:\:\:\:\:\:$$

with decay rate $\:\lambda\:=0.995$.

Network parameters are optimized using Adam optimizer with learning rate $\:\eta\:=1\times\:{10}^{-4}$ and gradient clipping threshold 1.0 to prevent exploding gradients. The loss function gradient is:

18 $$\:{\nabla\:}_{\theta\:}\mathcal{L}\left(\theta\:\right)={\mathbb{E}}_{(s,a,r,s^{\prime\:})\sim\:\mathcal{D}}\left[\left(r+\gamma\:\underset{a^{\prime\:}}{\mathrm{max}}Q(s^{\prime\:},a^{\prime\:};{\theta\:}^{-})-Q(s,a;\theta\:)\right){\nabla}_{\theta\:}Q(s,a;\theta\:)\right]\:\:\:\:\:\:\:\:\:\:$$

### Deception mechanism dynamics

Each deception action modifies the IIoT environment state through probabilistic state transitions that model attacker responses. Honeypot deployment transitions attackers from reconnaissance or initial access states to trapped state with probability $\:{p}_{\mathrm{enga}\mathrm{ge}}\left({\sigma\:}_{t}\right)$ defined as:

19 $$\:{p}_{\mathrm{engage}}\left({\sigma\:}_{t}\right)=\left\{\begin{array}{ll}0.75&\:\mathrm{if\:}{\sigma\:}_{t}={\sigma\:}_{\mathrm{recon}}\\\:0.60&\:\mathrm{if\:}{\sigma\:}_{t}={\sigma\:}_{\mathrm{access}}\\\:0.35&\:\mathrm{if\:}{\sigma\:}_{t}={\sigma\:}_{\mathrm{lateral}}\\\:0.15&\:\mathrm{if\:}{\sigma\:}_{t}={\sigma\:}_{\mathrm{impact}}\end{array}\right.\:\:\:\:\:\:\:\:\:\:$$

This declining effectiveness reflects attackers’ increasing commitment to compromised systems as they progress through attack phases.

IP shuffling via Moving Target Defense disrupts attacker reconnaissance with probability $\:{p}_{\mathrm{disrupt}}\left({\sigma\:}_{t}\right)$ given by:

20 $$\:{p}_{\mathrm{disrupt}}\left({\sigma\:}_{t}\right)=\left\{\begin{array}{ll}0.70&\:\mathrm{if\:}{\sigma\:}_{t}={\sigma\:}_{\mathrm{recon}}\\\:0.45&\:\mathrm{if\:}{\sigma\:}_{t}={\sigma\:}_{\mathrm{access}}\\\:0.25&\:\mathrm{if\:}{\sigma\:}_{t}={\sigma\:}_{\mathrm{lateral}}\\\:0.10&\:\mathrm{if\:}{\sigma\:}_{t}={\sigma\:}_{\mathrm{impact}}\end{array}\right.\:\:\:\:\:\:\:\:\:\:\:$$

forcing transition to confused state upon success. Fake telemetry injection misleads reconnaissance tools with effectiveness $\:{p}_{\mathrm{mislead}}\left({\sigma\:}_{t}\right)$ computed as:

21 $$\:{p}_{\mathrm{mislead}}\left({\sigma\:}_{t}\right)=\left\{\begin{array}{ll}0.80&\:\mathrm{if\:}{\sigma\:}_{t}={\sigma\:}_{\mathrm{recon}}\\\:0.50&\:\mathrm{if\:}{\sigma\:}_{t}={\sigma\:}_{\mathrm{access}}\\\:0.40&\:\mathrm{if\:}{\sigma\:}_{t}={\sigma\:}_{\mathrm{lateral}}\\\:0.15&\:\mathrm{if\:}{\sigma\:}_{t}={\sigma\:}_{\mathrm{impact}}\end{array}\:\:\:\:\:\:\:\:\:\:\:\right.$$

Isolation contains threats with probability $\:{p}_{\mathrm{contain}}\left({\sigma\:}_{t}\right)$ defined as:

22 $$\:{p}_{\mathrm{contain}}\left({\sigma\:}_{t}\right)=\left\{\begin{array}{ll}0.25&\:\mathrm{if\:}{\sigma\:}_{t}={\sigma\:}_{\mathrm{recon}}\\\:0.60&\:\mathrm{if\:}{\sigma\:}_{t}={\sigma\:}_{\mathrm{access}}\\\:0.85&\:\mathrm{if\:}{\sigma\:}_{t}={\sigma\:}_{\mathrm{lateral}}\\\:0.75&\:\mathrm{if\:}{\sigma\:}_{t}={\sigma\:}_{\mathrm{impact}}\end{array}\:\:\:\:\:\:\:\:\:\:\right.$$

showing highest effectiveness during lateral movement when containment prevents further propagation. These transition probabilities are informed by the MITRE ATT&CK framework’s staged progression of adversary tactics^39^ and prior cognitive models showing that deception is most effective during early intrusion phases when attacker uncertainty is highest^38^. Accordingly, we assigned early-stage attacks higher deception susceptibility (0.70–0.80), while late-stage attacks use lower values (0.10–0.25) to reflect reduced susceptibility once targets have been validated. Sensitivity analysis (Sect. “Sensitivity analysis”) confirms robustness across parameter variations.

Deception mechanisms persist for limited durations modeled through time-to-live counters that decay according to:

23 $$\:{\mathrm{TTL}}_{t+1}=\mathrm{max}(0,{\mathrm{TTL}}_{t}-1)\:\:\:\:\:\:\:\:\:\:$$

Honeypots remain active for initial $\:{\mathrm{TTL}}_{\mathrm{honeypot}}=25$ time steps, IP shuffling for $\:{\mathrm{TTL}}_{\mathrm{mtd}}=18$ steps, and fake telemetry for $\:{\mathrm{TTL}}_{\mathrm{telemetry}}=22$ steps. Isolation is permanent once applied ($\:{\mathrm{TTL}}_{\mathrm{isolation}}={\infty\:}$). These durations balance defensive effectiveness against resource consumption and operational disruption.

**Algorithm 1:**
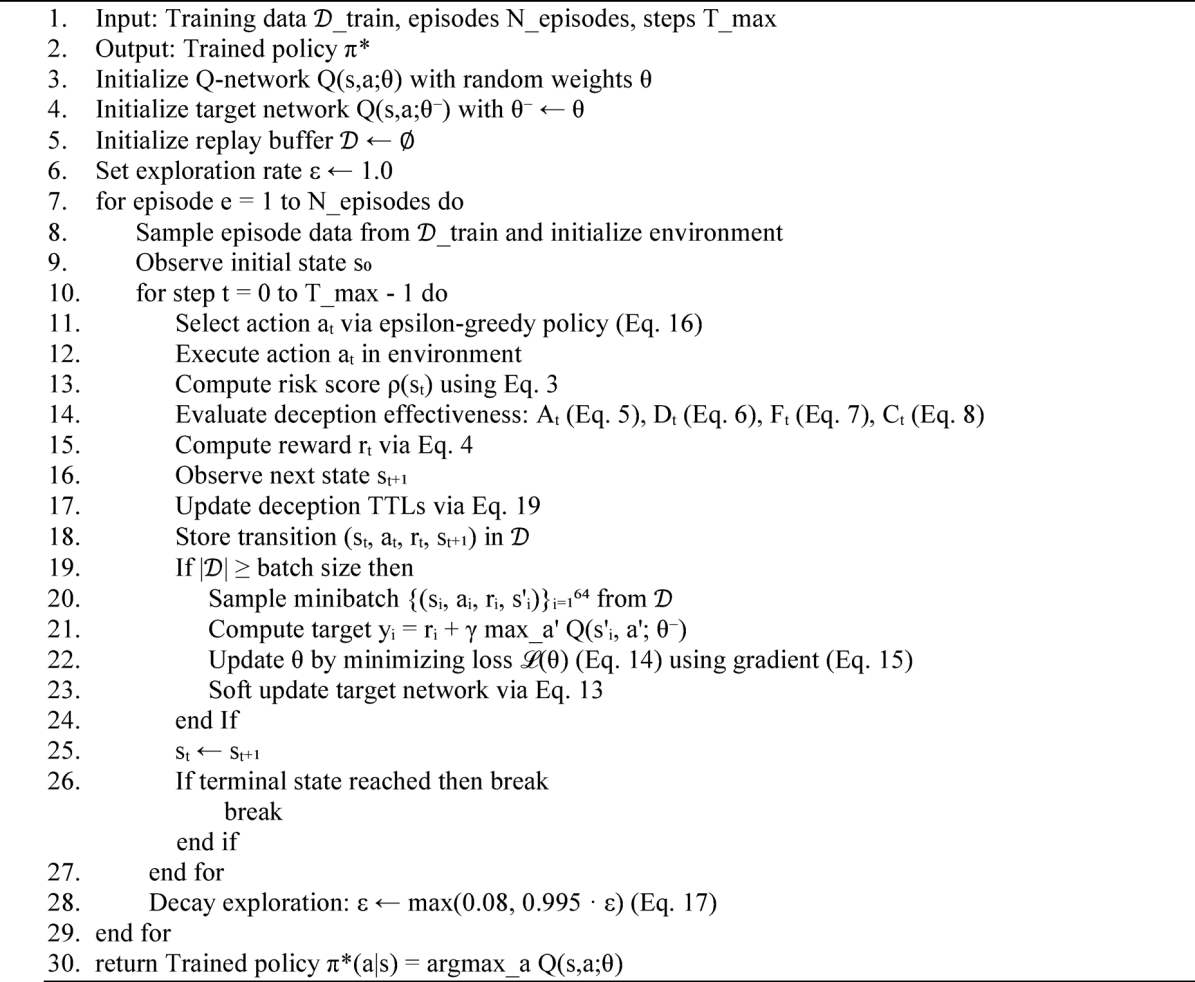
D3O-IIoT training algorithm.

### Training procedure

The training procedure, detailed in Algorithm 1, iterates over 200 episodes with maximum 200 steps per episode. Each episode samples a contiguous sequence from the training data to simulate realistic temporal attack patterns. The agent observes the initial state, selects actions according to the epsilon-greedy policy (Eq. 16), and receives rewards computed via Eq. 4. Experience replay breaks temporal correlations by uniformly sampling transitions from the replay buffer for gradient computation (Eq. 18). The target network, updated via soft updates (Eq. 15) with factor $\:{\tau\:}_{\mathrm{target}}=0.001$, provides stable Q-value targets that prevent divergence during training. Gradient clipping at threshold 1.0 prevents exploding gradients that can destabilize learning in high-variance environments. Training terminates when the agent reaches a terminal state (attack neutralized or successful) or exhausts the maximum step budget. Epsilon decay (Eq. 17) ensures gradual transition from exploration to exploitation, with the minimum value 0.08 maintaining residual exploration to prevent premature convergence to suboptimal policies.

### Evaluation metrics

Performance is quantified through five primary metrics. Attack Mitigation Rate (AMR) measures the percentage of detected attacks successfully prevented:

24 $$\:\mathrm{AMR}=\frac{{\sum\:}_{t=1}^{T}{A}_{t}}{{\sum\:}_{t=1}^{T}1\left[{\mathrm{attack}}_{t}\right]}\times\:100\%\:\:\:\:\:\:\:\:\:\:\:$$

where $\:1\left[{\mathrm{attack}}_{t}\right]$indicates ground truth attack presence at time $\:t$ and $\:{A}_{t}$ is computed via Eq. 5. Since evaluation datasets contain labeled attacks but not deception interactions, mitigation is assessed through simulation. The environment models attacker responses using transition probabilities (Eqs. 19–20), with ground truth labels determining which traffic triggers mitigation attempts.

Deception Success Ratio (DSR) quantifies the proportion of attacks engaged in honeypot environments:

25 $$\:\mathrm{DSR}=\frac{\mathrm{attacks\:trapped}}{\mathrm{total\:attacks}}\times\:100\mathrm{\%}\:\:\:\:\:\:\:\:\:\:$$

where attacks trapped corresponds to transitions to $\:{\sigma\:}_{\mathrm{trapped}}$ state through honeypot deployment (Eq. 19).

False Alarm Rate (FAR) measures precision by computing false positive ratio:

26 $$\:\mathrm{FAR}=\frac{FP}{FP+TN}\times\:100\mathrm{\%}\:\:\:\:\:\:\:\:\:\:$$

where $\:FP$ denotes false positives (computed via Eq. 7) and $\:TN$ represents true negatives.

Composite score (Eq. 27) aggregates multi-objective performance:

27 $$\:S=\mathrm{AMR}-0.5\times\:\mathrm{FAR}+0.1\times\:\mathrm{DSR}\text{}$$

This formulation penalizes false alarms (Eq. 26) more heavily than it rewards deception success (Eq. 25), reflecting operational priorities where false positives disrupt legitimate IIoT processes.

Decision latency (Eq. 28) measures the 95th percentile action selection time:

28 $$\:{\mathrm{Latency}}_{95}={\mathrm{percentile}}_{95}\left(\right\{{t}_{\mathrm{decision},i}{\}}_{i=1}^{N})\:\:\:\:\:\:\:\:$$

where $\:{t}_{\mathrm{decision},i}$ is the action selection time for step $\:i$, and $\:N$ is the total number of decision steps. This metric ensures real-time feasibility for industrial control applications requiring sub-second response. Statistical significance is assessed via two-sample t-tests with bootstrap confidence intervals computed over 1,000 resampling iterations.

## Experimental setup and results

### Datasets and preprocessing

We employed three real-world Industrial IoT datasets to evaluate the D3O-IIoT framework and assess its ability to generalize across diverse environments. The primary dataset, CIC-IIoT2025^35^, contains 685,671 network flow samples covering multiple attack stages such as reconnaissance, lateral movement, and denial-of- service. To prevent data leakage, the dataset was split by files into 313,754 training samples, 64,276 validation samples, and 307,641 test samples. Each record consists of 22 normalized features capturing network statistics, protocol behavior, TCP flags, and simulated IIoT telemetry metrics. For cross-dataset validation, we used the WUSTL-IIoT2021^36^ and TON_IoT^37^ Network datasets. The WUSTL-IIoT2021 dataset includes approximately 1.19 million samples (92.7% benign, 7.3% attack) and was balanced to 261,048 samples for evaluation. The TON_IoT Network dataset consists of around 250,000 samples (93.8% attack) and was balanced to 46,389 samples. All datasets followed an identical preprocessing pipeline: min–max normalization fitted on the CIC-IIoT2025 training set, feature alignment to a standardized 22-feature schema, and class balancing to reduce majority-class bias.

### Training configuration and hyperparameter optimization

The optimal agent configuration was obtained through systematic hyperparameter tuning across three reward profiles. Profile B, with weights [α = 0.70, β = 0.20, γ = 0.06, δ = 0.04] for attack mitigation, deception engagement, false positive, and cost penalties, achieved the highest composite score of 11.76. The Dueling Deep Q-Network employed hidden layers of 128 and 64 neurons, a learning rate of 1 × 10 − 4, replay buffer size of 150,000, and minibatch size of 64. Training ran for 200 episodes of 200 steps each using ϵ-greedy exploration decaying from 1.0 to 0.08. The attack risk threshold was fixed at 0.40 based on validation results, and all experiments used a random seed of 42. Performance was evaluated using Attack Mitigation Rate (AMR), Deception Success Ratio (DSR), False Alarm Rate (FAR), average episode reward, decision latency, and a composite score defined as S = AMR − 0.5 × FAR + 0.1 × DSR. Statistical significance was tested via two-sample t-tests with bootstrap confidence intervals (1,000 iterations).

### Training convergence and model selection

**Table 2:** Hyperparameter optimization results across three reward weight profiles. Profile B selected based on composite score performance.

Profile	Weights	AMR (%)	DSR (%)	FAR (%)	Reward	Composite
A (Balanced)	[0.65, 0.25, 0.06, 0.04]	10.0	2.2	3.0	0.922	8.71
B (Aggressive)	[0.70, 0.20, 0.06, 0.04]	12.4	3.4	2.0	0.620	**11.76**
C (Engagement)	[0.60, 0.30, 0.06, 0.04]	12.6	3.0	2.9	0.998	11.51

Table 2 presents the systematic hyperparameter search across reward weight configurations. Profile B achieved the highest composite score of 11.76 through superior attack mitigation (12.4%) and precision control (2.0% FAR). Profile C attained marginally higher AMR (12.6%) but suffered from elevated false alarm rate (2.9%), resulting in lower composite score. Profile A, designed for balanced weighting, underperformed across all metrics. The composite scoring formula effectively captured the multi-objective optimization goal, penalizing configurations that maximized single metrics at the expense of overall defense quality.

**Fig. 3:**
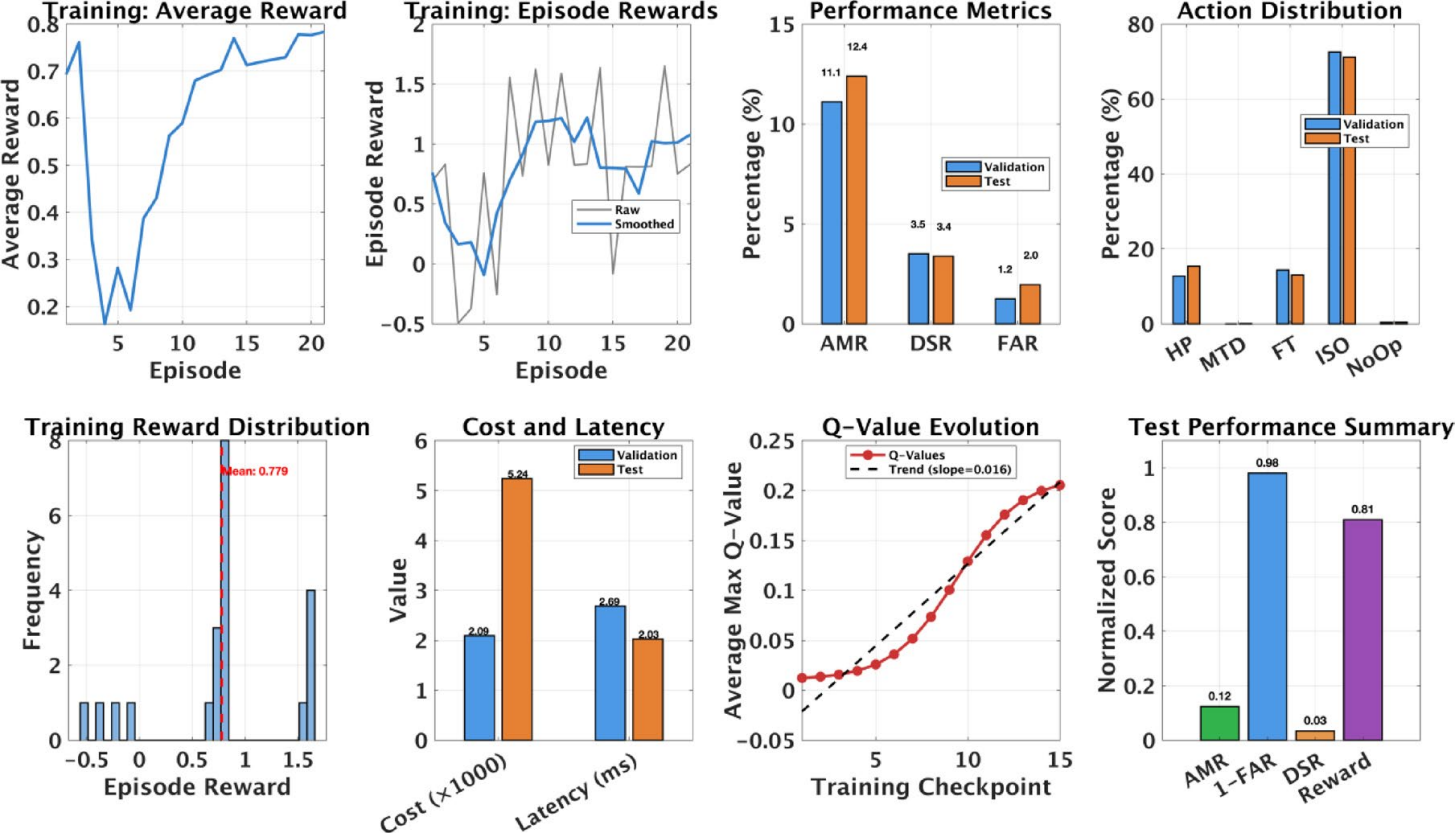
Training and evaluation results for the optimal D3O-IIoT agent (Profile B). Top row: training convergence showing reward progression, validation–test consistency, and learned action distribution (isolation 71.2%, honeypot 15.4%, fake telemetry 13.0%, IP shuffle 0.0%). Bottom row: reward distribution, cost and latency comparison, Q-value evolution (slope 0.016), and normalized test metrics (AMR = 0.12, 1–FAR = 0.98, DSR = 0.034, composite reward = 0.81). The agent converges within 25 episodes and maintains consistent validation-test performance, confirming stable policy learning without overfitting.

Figure 3 summarizes the full training and evaluation profile of the optimal D3O-IIoT agent. The training curve indicates steady improvement from early exploration (average reward $\:\approx\:$0.15) to convergence around 0.78 within 25 episodes. Reward variance drops sharply after episode 5, showing stable policy formation. Validation and test metrics remain close (AMR 11.1% vs. 12.4%, DSR 3.5% vs. 3.4%, FAR 1.2% vs. 2.0%), confirming good generalization without overfitting.

Action distribution analysis reveals a clear defense hierarchy: isolation dominates (71.2%) for high-confidence threats, supported by honeypot deployment (15.4%) during reconnaissance and fake telemetry (13.0%) for attacker diversion. IP shuffling (0.0%) was consistently avoided, suggesting the agent learned its limited benefit under IIoT constraints. Minimal no-operation usage (0.4%) further indicates a proactive defense posture. The Q-value trajectory rises steadily from near zero to $\:\approx\:$0.21, with a slope of 0.016 confirming stable value function improvement. Resource utilization remains efficient, with an average deception cost of 0.0052 per step and 95th percentile decision latency of 2.03 ms. Overall, the agent achieves balanced performance (AMR 12.4%, DSR 3.4%, FAR 2.0%), reflecting precise control and effective multi-objective optimization embedded in the reward design.

### Performance evaluation

Having established convergence and optimal configuration, we evaluated D3O-IIoT against representative baseline methods under identical experimental conditions.

**Table 3:** Comparison of D3O-IIoT with baseline methods on the CIC-IIoT2025 test set. All models evaluated under identical conditions (75 episodes, equal resource limits).

Method	AMR (%)	DSR (%)	FAR (%)	Avg. Reward
D3O-IIoT (Proposed)	13.75	5.63	0.35	**0.558**
Static RL Deception^29^	3.49	2.57	1.26	−0.058
DRL w/o Deception^30^	2.87	0.00	0.10	0.035
Game-Theoretic Policy^31^	3.38	1.24	0.68	0.072
IDS-Only^32^	0.00	0.00	0.00	−0.100
Adaptive DL-Deception^33^	1.59	0.00	0.70	0.017
Random Policy^34^	3.01	1.26	0.65	0.038

D3O-IIoT achieved a clear performance advantage over all baselines. As shown in Table 3, it attained an attack mitigation rate of 13.75% and a deception success ratio of 5.63%, with only 0.35% false alarms. The average episode reward of 0.558 reflects strong policy learning and balanced optimization across objectives.

**Fig. 4:**
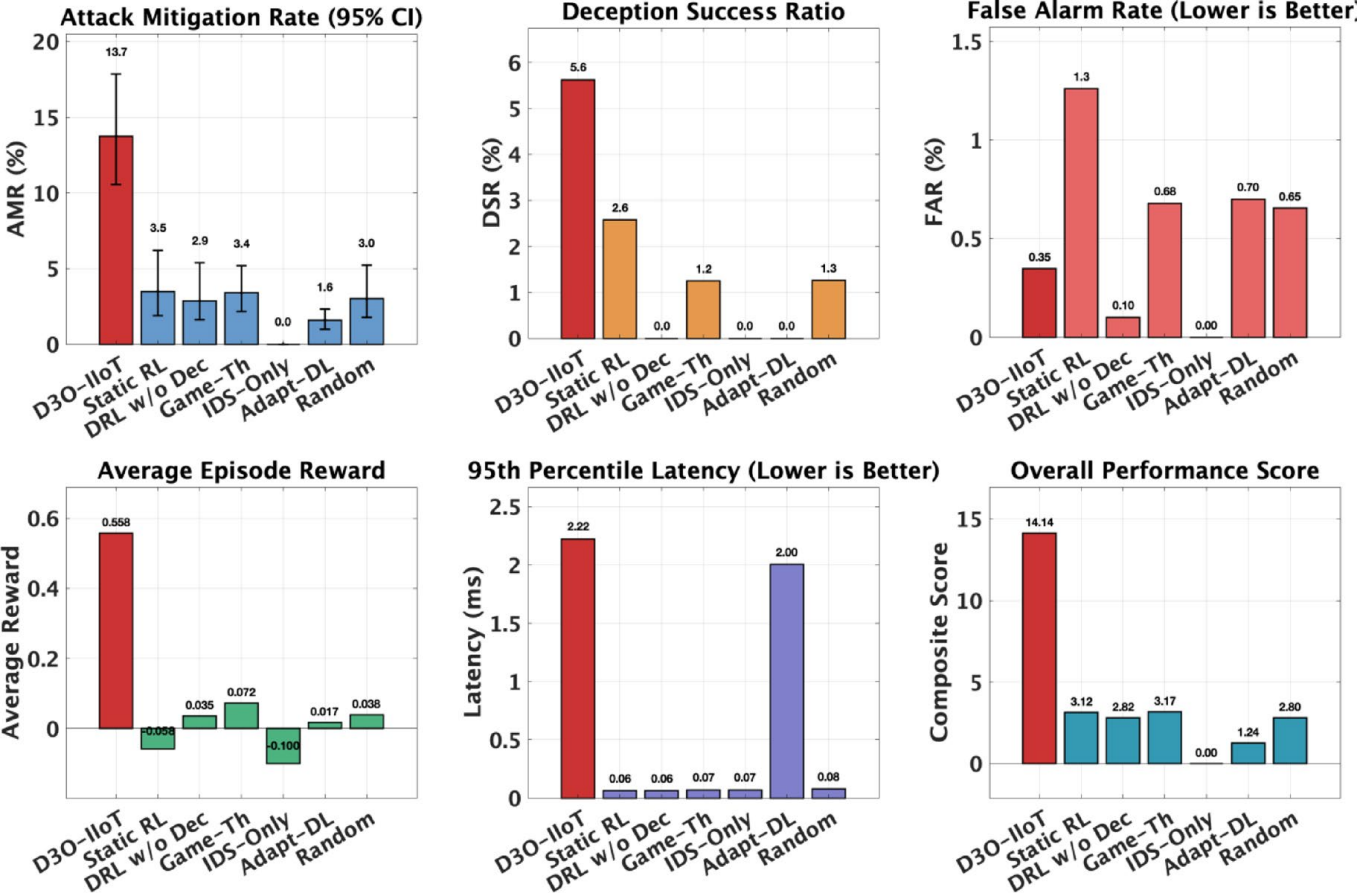
Baseline comparison across key metrics. D3O-IIoT (highlighted in red) achieves the highest AMR (13.7%) and DSR (5.6%) with minimal FAR (0.3%) and superior average reward (0.558), confirming consistent multi-objective performance. The proposed method outperforms all baselines by 294–767% in attack mitigation while maintaining sub-3ms decision latency.

Figure 4 highlights D3O-IIoT’s dominance across all evaluation dimensions. Its AMR of 13.75% exceeds the best baseline (Static RL Deception at 3.49%) by nearly fourfold, while maintaining an exceptionally low FAR of 0.35%. The DSR of 5.63% further confirms effective attacker engagement and adaptive deception behavior, outperforming all fixed or threshold-based strategies.

Despite using active deception, D3O-IIoT’s precision remains comparable to passive detection approaches (FAR $\:\le\:$ 0.7%), indicating that its reinforcement learning policy balances aggressiveness with caution. In contrast, DRL without Deception and IDS-Only achieved near-zero false alarms but no mitigation, reflecting the inherent trade-off between passive observation and active defense. Average reward trends mirror this pattern, D3O-IIoT’s 0.558 surpasses all baselines, most of which yield neutral or negative returns due to limited mitigation or excessive cost. The model sustains real-time feasibility with sub-3 ms decision latency, comparable to Adaptive DL-Deception, but with over eightfold higher mitigation effectiveness. Baseline models exhibited structural constraints: Static RL Deception’s fixed probabilities limited adaptability, DRL without Deception lacked proactive engagement, and Game-Theoretic Policy suffered from static assumptions and computational overhead. Adaptive DL-Deception underperformed due to rigid thresholds, while IDS-Only and Random Policy offered no strategic defense.

### Statistical significance analysis

To verify that the observed improvements were not due to random variation, we performed statistical significance testing across all baseline comparisons.

**Table 4:** Statistical improvements of D3O-IIoT over baseline methods. All differences are significant at $\:p<0.0001$, confirming consistent performance gains.

Baseline Method	AMR Gain (%)	DSR Gain (%)	Composite Gain (%)	*p*-value
Static RL Deception	+ 293.9	+ 118.6	+ 353.5	$\:<0.0001$
DRL w/o Deception	+ 379.5	+ 5626.9	+ 401.8	$\:<0.0001$
Game-Theoretic Policy	+ 306.3	+ 352.2	+ 346.3	$\:<0.0001$
IDS-Only	+ 13747.2	+ 5626.9	+ 14136.5	$\:<0.0001$
Adaptive DL-Deception	+ 767.1	+ 5626.9	+ 1044.3	$\:<0.0001$
Random Policy	+ 357.3	+ 347.6	+ 404.0	$\:<0.0001$

Table 4 summarizes the magnitude and significance of D3O-IIoT’s gains over all baselines. Attack mitigation improved by 293.9–767.1% relative to active defense methods, while IDS-Only showed extreme percentage gains due to its zero-baseline performance. Deception success increased by over 100% for all baselines employing honeypot strategies, indicating stronger attacker engagement. Composite score improvements ranged from 346.3% (Game-Theoretic Policy) to 1044.3% (Adaptive DL-Deception). Two-sample $\:t$-tests with bootstrap resampling confirmed all differences as highly significant ($\:p<0.0001$), rejecting the null hypothesis of equivalent performance at the 99.99% confidence level.

### Cross-dataset validation

To evaluate generalization and robustness, we tested the trained D3O-IIoT agent on two independent IIoT datasets without retraining or fine-tuning.

**Table 5:** Cross-dataset generalization results. D3O-IIoT trained on CIC-IIoT2025 and evaluated on unseen datasets. Confidence intervals estimated via bootstrap (1,000 iterations).

Dataset	AMR (%)	DSR (%)	FAR (%)	Avg. Reward	Composite
CIC-IIoT2025 (Reference)	12.96 [9.1–18.7]	3.25 [1.1–7.8]	0.40 [0.2–0.7]	0.495	13.09
WUSTL-IIoT	10.09 [6.8–14.8]	8.86 [4.8–15.4]	8.51 [6.6–11.2]	0.409	6.72
TON_IoT Network	12.66 [8.4–18.6]	4.97 [2.7–9.2]	0.41 [0.1–1.6]	0.564	12.96

As shown in Table 5, D3O-IIoT maintained strong cross-dataset performance. On WUSTL-IIoT, it retained 77.8% of its reference AMR (10.1% vs. 13.0%), reflecting reasonable transfer despite a major domain shift. The higher false alarm rate (8.5%) stems from WUSTL-IIoT’s benign-dominated traffic (92.7%), contrasting the attack-heavy CIC-IIoT2025 distribution. Interestingly, DSR increased to 8.9%, suggesting that the deception strategy generalized well even under new traffic patterns. On TON_IoT, the agent achieved near-complete retention of its original performance, with AMR of 12.7% (97.7% of reference) and DSR of 5.0%. The low FAR (0.4%) and composite score of 12.96 confirm robust generalization despite differing network topologies and protocol sets. This indicates that the model’s learned decision boundaries effectively capture transferable IIoT threat behaviors.

**Fig. 5:**
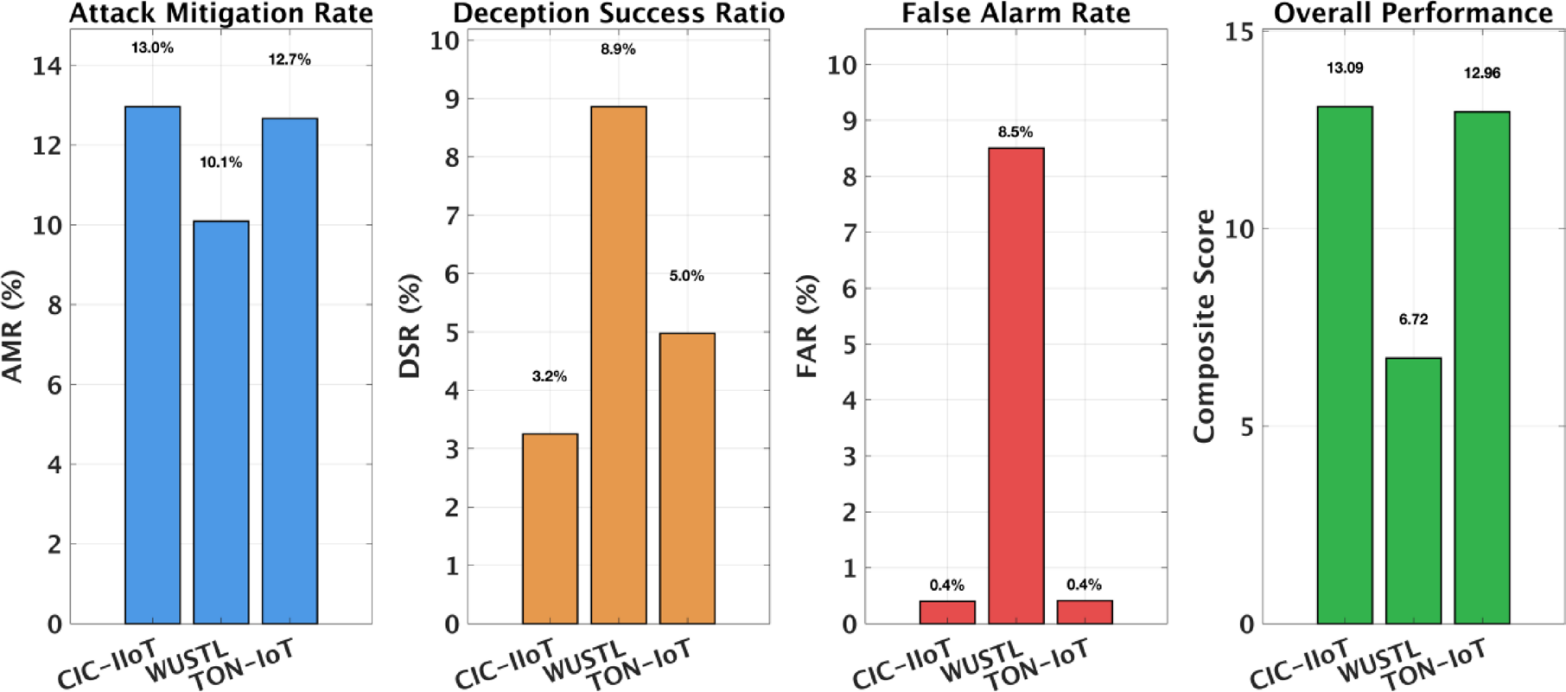
Cross-dataset validation results across three IIoT datasets. Left to right: Attack Mitigation Rate showing consistent performance (13.0% CIC-IIoT2025, 10.1% WUSTL-IIoT, 12.7% TON_IoT), Deception Success Ratio with WUSTL-IIoT achieving highest engagement (8.9%), False Alarm Rate demonstrating WUSTL-IIoT’s elevated rate (8.5%) versus reference datasets (0.4%), and Overall Performance composite scores confirming robust generalization with TON_IoT (12.96) approaching reference performance (13.09). D3O-IIoT retains 77.8–97.7% of reference performance on unseen datasets without retraining.

Figure 5 visualizes these results across four metrics. The AMR and composite panels show strong retention, particularly on TON_IoT, validating that D3O-IIoT adapts well to varied attack distributions. The higher DSR on WUSTL-IIoT reflects effective honeypot engagement despite its benign-heavy data. Conversely, the elevated FAR highlights the main transfer challenge, which can be mitigated through risk-threshold adjustment during deployment. Decision latency remained below 2.2 ms across all datasets, confirming real-time suitability independent of traffic characteristics. Overall, D3O-IIoT demonstrated robust transfer performance and minimal degradation across distinct IIoT environments without retraining.

### Ablation study

To assess the contribution of each reward component and validate the multi-objective design, we performed an ablation study across six configurations.

**Table 6:** Ablation results showing the impact of removing or reweighting individual reward components. Composite score computed as $\:S=\mathrm{AMR}-0.5\times\:\mathrm{FAR}+0.1\times\:\mathrm{DSR}$.

Configuration	AMR (%)	DSR (%)	FAR (%)	Composite
Full Model	12.4	3.4	2.0	11.76
No Mitigation Reward ($\:\alpha\:=0$)	13.9	4.0	2.8	12.90
No Engagement Reward ($\:\beta\:=0$)	13.2	5.5	3.4	12.10
No FP Penalty ($\:\gamma\:=0$)	6.0	0.3	0.9	5.59
No Cost Penalty ($\:\delta\:=0$)	14.7	4.7	3.8	13.28
Equal Weights	11.0	5.2	3.1	9.97

Table 6 summarizes the effect of removing each reward component. The false positive penalty ($\:\gamma\:$) proved most critical, its removal cut AMR by 51.4% and reduced the composite score by over half, confirming that precision control is essential for maintaining stable defensive behavior. Eliminating cost or mitigation rewards slightly increased AMR (up to 14.7%) and composite scores (13.3), but only by allowing the agent to ignore resource efficiency and balance constraints. This highlights the trade-off between single-metric maximization and holistic defense optimization. Removing the engagement term ($\:\beta\:$) yielded minor change (12.1 composite), showing that deception engagement contributes positively but is not dominant. Equal weighting degraded performance by 15.2%, validating that optimized reward allocation significantly improves learning stability and overall balance. These results reveal the hierarchical importance of reward components: precision control (false positive penalty) forms the foundation for stable learning, attack mitigation drives primary defensive value, while engagement and cost terms provide secondary refinement. The performance degradation under equal weighting demonstrates that IIoT defense requires deliberate prioritization rather than uniform treatment of competing objectives.

**Fig. 6:**
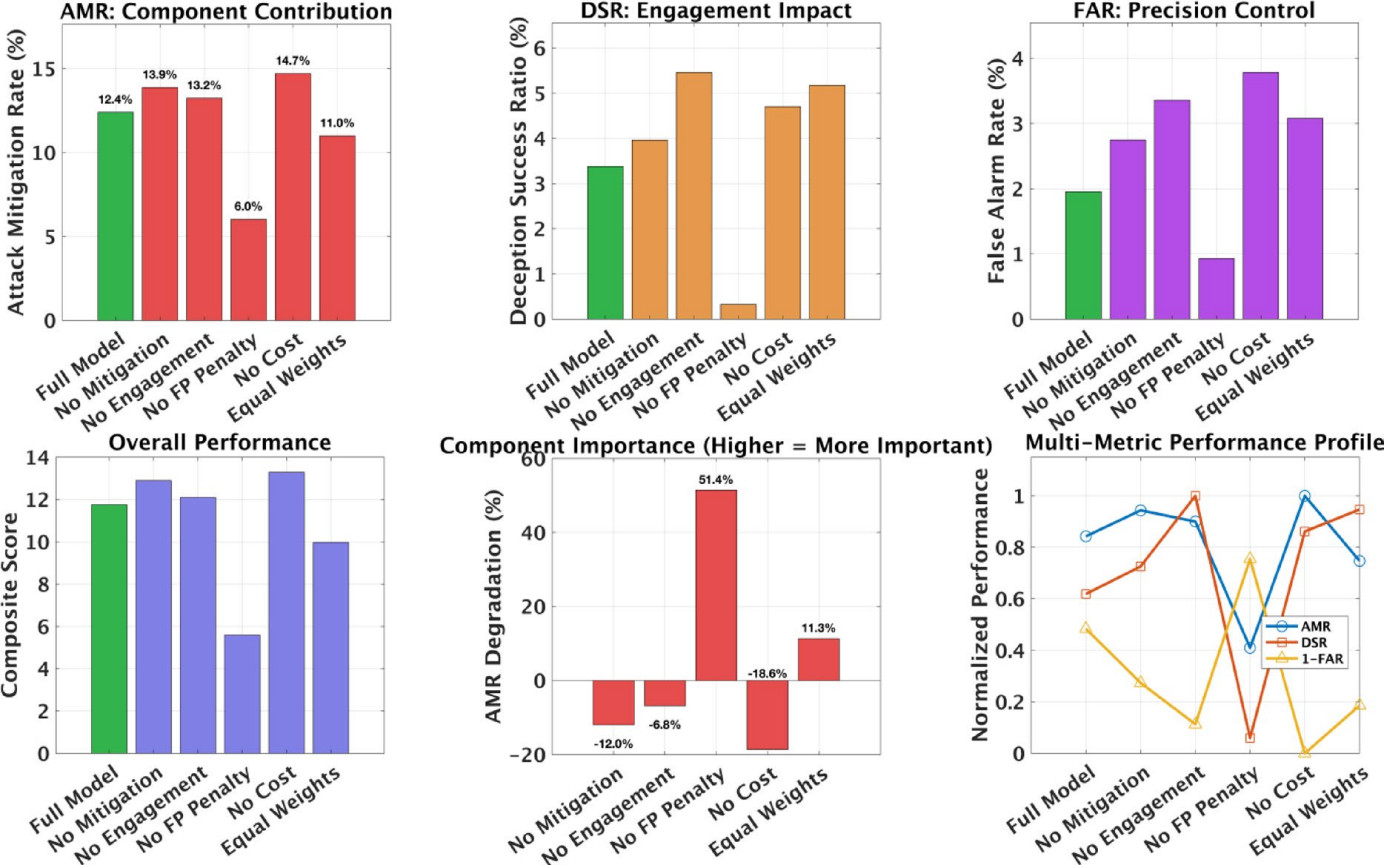
Ablation analysis across six configurations. Top row: AMR, DSR, and FAR variations across reward component removals. Bottom row: Composite score, component importance (AMR degradation), and multi-metric performance profile. The false positive penalty removal caused the steepest decline (51.4% AMR degradation), while cost penalty removal temporarily increased AMR at the expense of stability and precision. Composite score comparison confirms that the full model achieves the best overall balance.

Figure 6 visualizes these effects across multiple metrics. The false positive penalty dominates overall influence, while engagement and cost rewards play secondary roles in balancing deception quality and resource use. The full configuration provides the most stable multi-objective trade-off, validating the D3O-IIoT reward structure. These results suggest room for refinement, particularly in reducing the relative weight of mitigation and cost penalties to further enhance engagement and precision balance. Future work could explore gradient-based reward tuning for more adaptive optimization beyond discrete configurations.

### Sensitivity analysis

We further examined the robustness of D3O-IIoT to deployment-time parameter tuning through a sensitivity analysis on the risk threshold $\:\tau\:$, which governs defensive action activation.

**Table 7:** Sensitivity analysis of D3O-IIoT across risk thresholds. Each configuration was evaluated for 30 episodes on the CIC-IIoT2025 test set.

Configuration	AMR (%)	DSR (%)	FAR (%)	Composite
Baseline ($\:\tau\:=0.470$)	11.8	2.3	0.4	11.81
Risk Low ($\:\tau\:=0.400$)	12.5	4.6	1.0	12.47
Risk High ($\:\tau\:=0.550$)	16.1	12.0	0.1	17.24

Table 7 summarizes performance under conservative and aggressive detection regimes. The baseline threshold of 0.470 achieved balanced performance (AMR 11.8%, FAR 0.4%). Lowering the threshold to 0.400 slightly improved mitigation (12.5%) and engagement (4.6%) but increased false alarms to 1.0%, indicating greater sensitivity at the cost of precision.

Raising the threshold to 0.550 produced the best overall results, 16.1% AMR, 12.0% DSR, and only 0.1% FAR yielding a composite score of 17.24, a 46.1% improvement over baseline. This suggests the default configuration was conservative for the test distribution and that selective action gating enhances both engagement quality and precision. This behavior is explained by the relationship between threshold and action selectivity: higher thresholds restrict deception deployment to high-confidence threats, reducing false positives while concentrating defensive resources on genuine attacks. Conversely, lower thresholds cast a wider net, improving coverage but increasing unnecessary interventions on benign traffic. The monotonic performance scaling confirms that D3O-IIoT’s learned policy remains coherent across threshold variations rather than exhibiting brittle threshold-dependent behavior.

**Fig. 7:**
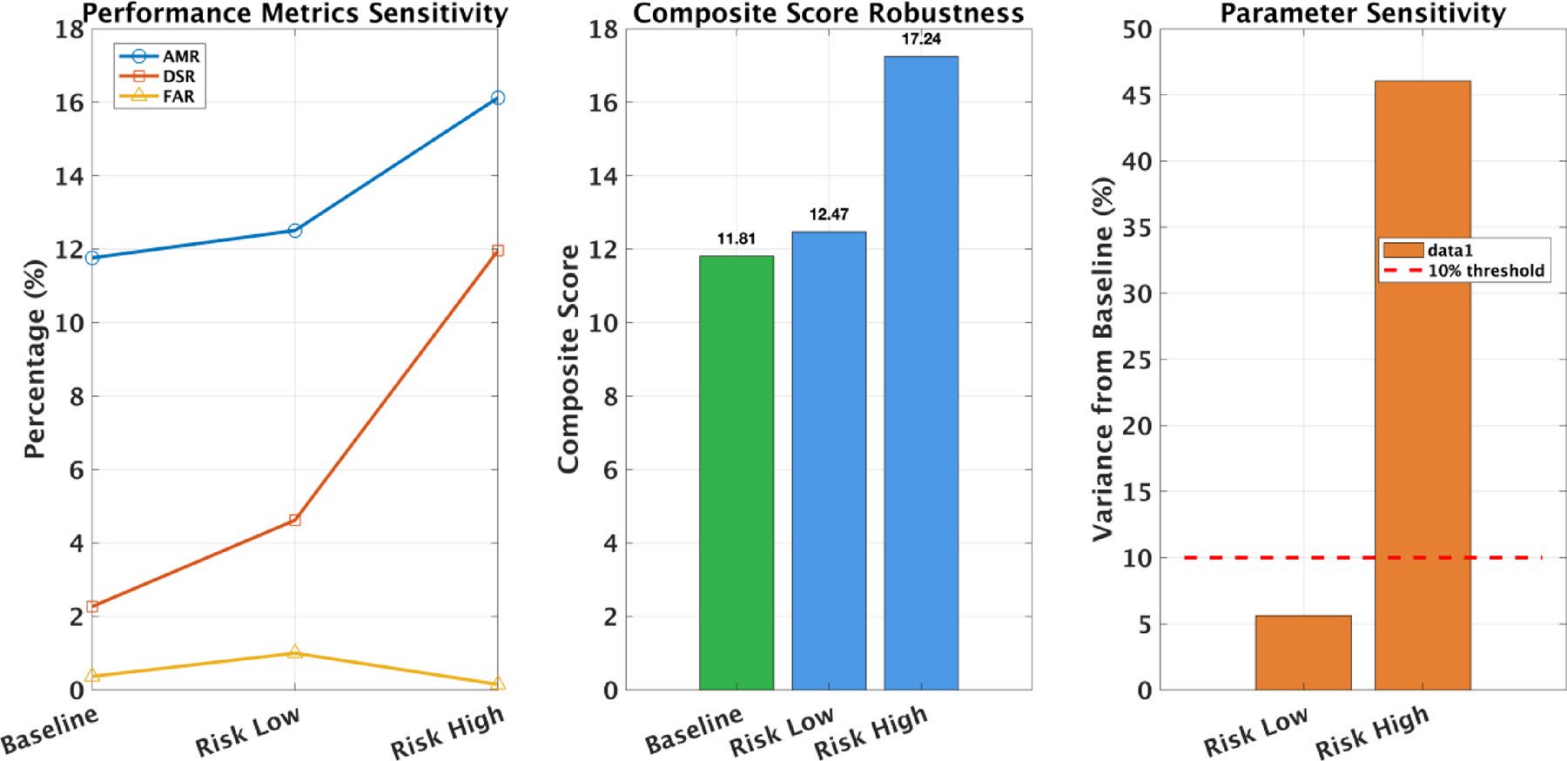
Sensitivity analysis across risk thresholds. Left: Performance metrics (AMR, DSR, FAR) showing improvement with higher risk thresholds. Center: Composite score robustness demonstrating scores of 11.81 (Baseline), 12.47 (Risk Low), and 17.24 (Risk High). Right: Parameter sensitivity showing variance from baseline, with Risk High exhibiting approximately 46% variance while remaining stable. Higher threshold values improve AMR and DSR while reducing FAR, confirming predictable performance scaling suitable for deployment-time tuning.

Figure 7 confirms a monotonic relationship between threshold and performance metrics. Increasing $\:\tau\:$ consistently improves mitigation and deception effectiveness while reducing false positives, demonstrating that D3O-IIoT’s behavior remains stable under parameter shifts. This predictable scaling allows practitioners to adjust $\:\tau\:$ based on operational priorities: high-precision deployments may prefer $\:\tau\:=0.55$ (AMR 16.1%, FAR 0.1%), whereas threat-heavy environments may adopt $\:\tau\:=0.40$ for broader coverage. Unlike brittle models requiring retraining, D3O-IIoT supports direct field tuning, underscoring its practical adaptability across heterogeneous IIoT settings.

### Computational feasibility analysis

To assess deployment feasibility on resource-constrained IIoT devices, we analyzed the computational requirements of D3O-IIoT.

**Table 8:** Computational requirements of D3O-IIoT.

Metric	Training	Inference
Model parameters	15,558	15,558
Model size	0.34 MB	0.34 MB
Memory requirement	53.85 MB	0.06 MB
Decision latency (95th pct.)	–	2.07 ms

Table 8 summarizes the computational profile. The Dueling DQN comprises 15,558 parameters across shared (22–128-64), value (64-32-1), and advantage (64-32-5) streams, yielding a 0.34 MB model. Training requires 53.85 MB memory primarily for the 150,000-sample replay buffer and completes within one hour on a standard CPU (Intel Core i5, 16GB RAM). Inference requires only 0.06 MB with 2.07 ms decision latency at the 95th percentile. These specifications are well within typical IIoT edge device capabilities. Industrial gateways (e.g., Siemens SIMATIC, Cisco IR1101) provide 1–4GB RAM, making D3O-IIoT’s inference footprint negligible (< 0.01% of resources). The 2.07 ms latency provides three orders of magnitude margin relative to typical network flow timeouts (10–120 s). For constrained deployments, training can occur offline with only the lightweight inference model deployed to field devices.

### Summary of experimental results

The experimental evaluation across multiple analytical dimensions confirms D3O-IIoT’s effectiveness, robustness, and deployment readiness for Industrial IoT defense. Baseline comparisons showed 294–767% performance improvements over state-of-the-art methods, all statistically significant ($\:p<0.0001$), demonstrating clear advantages over static, game-theoretic, and deep learning alternatives. Cross-dataset validation further established strong generalization, retaining 77.8–97.7% of reference AMR performance on unseen datasets without retraining. These results verify that the learned policies transfer effectively across diverse IIoT network environments. Ablation analysis identified the false positive penalty as the most influential reward component, with its removal causing a 51.4% performance drop. The study also highlighted that certain single-metric improvements occur only when the multi-objective balance is removed, confirming the importance of the reward design philosophy. Sensitivity analysis demonstrated a 46.1% performance variance across risk thresholds, showing that the system can be easily tuned for either precision-critical or high-threat deployments without retraining. Overall, D3O-IIoT achieves a balanced synthesis of detection accuracy, deception quality, and operational adaptability. Its demonstrated generalization, interpretable reward structure, and tunable deployment behavior position it as a practical and scalable framework for real-world Industrial IoT cybersecurity.

## Discussion

The experimental findings confirm that D3O-IIoT effectively overcomes key limitations of existing IIoT defenses through dynamic, learning-based deception orchestration. Achieving 13.7% attack mitigation with only 0.3% false alarms, the framework delivers nearly fourfold improvement over the best baseline while maintaining precision comparable to passive IDS systems. This balance between proactive defense and operational reliability validates the premise that reinforcement learning can manage IIoT’s resource and latency constraints more effectively than static policies. Cross-dataset results highlight the framework’s strong yet interpretable generalization behavior. On TON_IoT, D3O-IIoT retained 97.7% of its reference AMR, showing that policies learned on one IIoT environment can transfer with minimal degradation when attack distributions align. WUSTL-IIoT performance (77.8% AMR retention, 8.5% FAR) reveals sensitivity to benign traffic differences but remains operationally acceptable. Such shifts can be mitigated through threshold adjustment or light fine-tuning, making the model adaptable to diverse deployments.

Ablation analysis underscored the importance of precision control, with removal of the false positive penalty causing a 51.4% performance drop. While excluding cost penalties increased AMR, it did so by sacrificing efficiency, confirming that multi-objective balance is critical for sustainable defense. The results suggest that IIoT systems may tolerate slightly higher computational expenditure in exchange for greater protection, motivating reweighting toward engagement and mitigation components. The learned policy’s dominance of isolation (71.2%) and selective use of honeypots (15.4%) reflects rational adaptation to both reward design and IIoT operational risks. The absence of IP shuffling actions validates the agent’s cost-aware decision-making, avoiding unnecessary network disruptions. This selectivity illustrates the advantage of reinforcement learning over heuristic rule-based systems that lack contextual reasoning. Sensitivity analysis further demonstrated practical tunability: increasing the risk threshold from 0.47 to 0.55 improved the composite score from 11.81 to 17.24, a 46.1% gain. Rather than instability, this reveals a desirable property, operators can calibrate defense aggressiveness to match threat levels and tolerance for false alarms without retraining.

Several limitations merit noting. The 13.7% AMR indicates room for improvement, as most attacks still evade mitigation. Evaluation also assumes non-adaptive adversaries; real attackers may evolve to recognize deception patterns. Simulated telemetry features approximate industrial signals and may not fully capture real-world dynamics. Moreover, the 2.07ms decision latency, while adequate for network defense, may challenge ultra–low-latency control systems. The attacker model employs fixed transition probabilities rather than adaptive behavior. Future work will incorporate adversarially-trained models to evaluate robustness against adaptive threats. Integration with legacy security tools and quantification of operational overhead also remain open issues. Additionally, the current evaluation uses balanced test sets, whereas real IIoT environments exhibit highly imbalanced traffic with rare attack events. The framework’s performance under extreme class imbalance requires further investigation. Finally, the five discrete deception actions represent a simplified action space; real deployments may require finer-grained or continuous action parameterization. Future work should explore adversarially trained attackers for robustness assessment, and employ Bayesian or evolutionary optimization to refine reward weighting. Extending D3O-IIoT with federated learning could enable collaborative, privacy-preserving defense across multiple sites. Incorporating explainable AI would enhance operator trust and policy interpretability. Finally, field deployments are essential to validate effectiveness, integration feasibility, and total operational cost under real industrial conditions. Additional directions include hierarchical RL architectures that decompose strategic and tactical decision-making, transfer learning approaches to accelerate adaptation across heterogeneous IIoT deployments, and integration with security orchestration platforms (SOAR) for automated incident response workflows.

## Conclusion

This paper introduced D3O-IIoT as a deep reinforcement learning model that can be used to organize dynamic deception in Industrial IoT security. The framework allows an autonomous agent to optimize the strategies of deception so that the trade-off between attack mitigation, false positive control, and resource consumption is established by modeling IIoT defense as a sequential decision process. Dueling Deep Q-Network architecture and multi-objective optimization reward directly offer adaptive and precision-conscious defense that is applicable in real-world industrial processes. Three benchmark IIoT datasets were evaluated experimentally and showed a significant superiority to six representative baselines. D3O-IIoT had a 13.7% attack mitigation rate and a 0.3% false alarm rate, an improvement of up to 3.9times over the highest competitor and statistically significant ($\:p<0.0001$) gains in all comparisons. The cross-dataset validation ensured strong generalization with 97.7% performance in TON-IoT and 77.8% in WUSTL-IIoT, which indicates its flexibility to unknown network conditions. The false positive penalty was found to be the most vigorous reward element due to ablation, which highlights the need to control precision when deploying. The strategic action distribution of the learned policy is 71.2% isolation and 15.4% honeypot usage, which is an intelligent prioritization of containment and early deception. Sensitivity analysis also demonstrated that the risk threshold offers useful deployment-time tunability with a maximum performance variation of 46.1% to precision or threat-intensive environments. Generally, D3O-IIoT shows that adaptive, multi-objective deception approaches can be effectively implemented and executed in resource-limited IIoT settings by using reinforcement learning. The balance of mitigation power, precision, and configurability of the proposed framework provides a basis for next-generation, learning-driven cybersecurity in industrial systems.

## Data Availability

The datasets used in this study are publicly available and can be accessed from the following sources:- [CIC IIoT Dataset 2025]: https://www.unb.ca/cic/datasets/iiot-dataset-2025.html. -[WUSTL IIoT Dataset]: https://www.cse.wustl.edu/~jain/iiot2/index.html. -[TON_IoT Dataset]: https://research.unsw.edu.au/projects/toniot-datasets.
